# Follow-up and prognosis of HCM

**DOI:** 10.21542/gcsp.2018.33

**Published:** 2018-08-12

**Authors:** Fernando Dominguez, Jorge Sanz-Sánchez, Pablo García-Pavía, Esther Zorio

**Affiliations:** 1 Department of Cardiology, Hospital Universitario Puerta de Hierro Majadahonda, Manuel de Falla, Majadahonda, Madrid, Spain; 2 Hospital Universitario y Politécnico La Fe, Valencia, Spain

## Introduction

The frequency of follow-up visits in hypertrophic cardiomyopathy (HCM) patients is mainly determined by their symptoms, age and severity of disease. Clinical visits should focus on sudden cardiac death (SCD) and embolic risk-assessment, changes in symptoms, cardiac rhythm, left ventricular outflow tract obstruction (LVOTO) and left ventricular (LV) systolic function^1^.

## Patients with HCM phenotype

In asymptomatic patients, a clinical examination with 12-lead electrocardiogram (ECG) and transthoracic echocardiography should be preferably performed on a yearly basis or every two years at most^1^. 24-hour Holter monitoring is recommended every 1–2 years or every 6 months in patients with left atrial (LA) dilation of more than 45 mm in order to detect asymptomatic atrial fibrillation that would warrant oral anticoagulation therapy^1^. The rationale for this specific threshold is that it has been observed that the risk of thromboembolism rises exponentially with LA diameter above 45–50 mm^2^.

Indication for implanted cardioverter-defibrillator (ICD), based on the HCM risk-score, should be re-evaluated in each follow-up visit^3^, as well as indication for oral anticoagulation.

Symptomatic patients with LVOTO need closer follow-up every 3–6 months in order to evaluate response to medical treatment and to plan eventual septal reduction therapies. Once an invasive reduction therapy is performed, a clinical follow-up including ECG, transthoracic echocardiogram and ambulatory Holter monitoring should be performed at 3 and then at 6–12 months^1^. Additionally, cardiac magnetic resonance imaging (MRI) evaluation could be considered every 2–3 years^1^, especially when clinical progression is detected or to decide ICD indication in borderline and doubtful cases, given the prognostic value of late gadolinium enhancement (LGE)^4^. Also a cardiopulmonary exercise test is a valuable tool to objectively assess functional capacity once heart failure (HR) is present. In a large study with HCM patients who underwent cardiopulmonary exercise test, peak oxygen consumption, ventilatory efficiency and ventilatory anaerobic threshold were found to be predictors of death and heart transplant^5^, so this tool can be useful in providing prognostic information.

**Figure 1.**
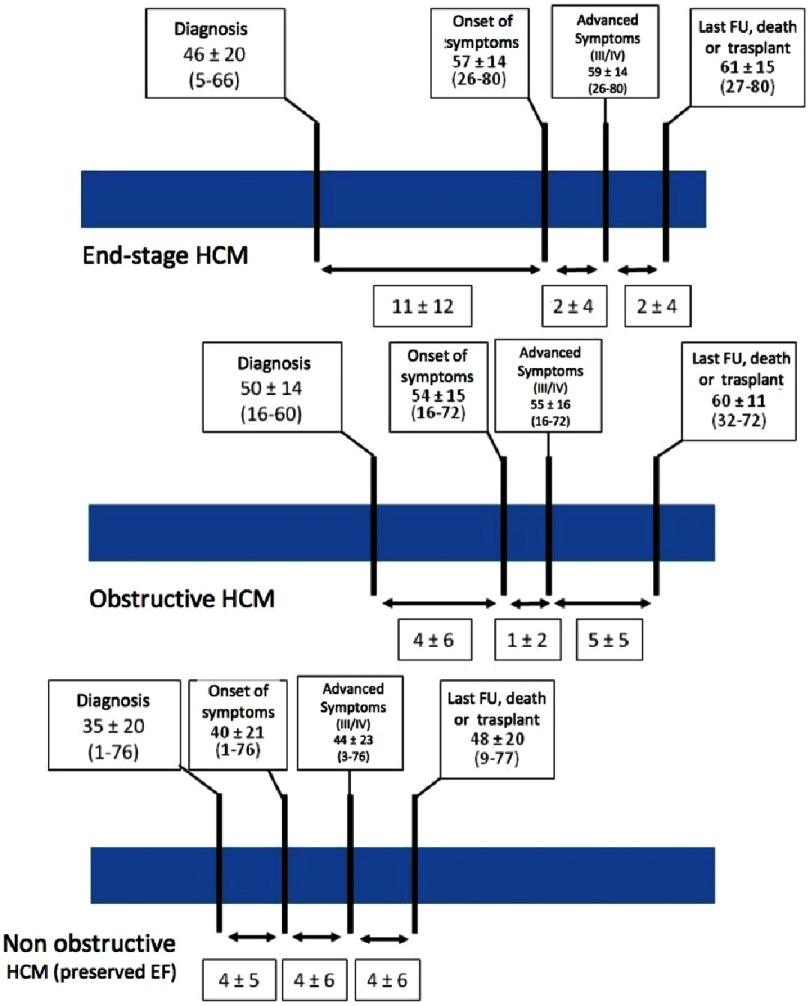
Time lines for the three different hypertrophic cardiomyopathy phenotypes. Patient ages at intervals describe clinical evolution. FU, follow-up. Reproduced with permission from Melacini P et al. Eur Heart J. 2010; 31: 2111–2123.^6^

Patients with non-obstructive HCM also present symptoms due to diastolic dysfunction or microvascular angina, and have an earlier onset of HF and a more rapid progression to advanced HF and adverse outcome as compared to patients with LVOTO^6^. The average time between diagnosis and onset of HF is reported to be 11 years, but once HF is present only a mean period of 4 years is necessary until death or heart transplantation (Figure 1).

## Mutation carriers without a phenotype

Although data regarding follow-up in mutation carriers without a phenotype are scarce, a recent study from Rotterdam has shown that during a mean follow-up period of 7 years 16% of genotype-positive individuals develop left ventricular hypertrophy (LVH) consistent with HCM diagnosis with survival at 5/10 years of 99%/95%^7^. Other recent studies also suggest that clinical course in most unaffected mutation carriers is benign^1,8^. Clinical screening with ECG and echocardiography in first degree relatives of HCM patients can start during childhood or up to 10–12 years^9^, as clinically important events in asymptomatic children are very rare before puberty^8^.

The recommended intervals for follow-up are different in European and American HCM guidelines. The former do not establish a specific interval but recommend a long-term evaluation in normal healthy mutation carriers^1^. The American College of Cardiology Foundation/American Heart Association (ACCF/AHA) recommends screening at least every 5 years above the age of 21 and every 12-18 months from age 12 to 18-21^10^. Moreover, a position statement from the European Society of Cardiology (ESC) working group on myocardial and pericardial diseases established two possible scenarios in the follow-up of mutation carriers without HCM phenotype: causal mutation identified or not in the probands with HCM phenotype. In the first case, phenotype negative relatives who are proved to be non-carriers do not require further cardiac evaluation and can be discharged. On the other hand, mutation carriers could benefit from an initial cardiac MRI apart from ECG and echocardiogram in order to assess early signs of HCM and a yearly follow-up is recommended between the age of 10 and 20, and then every 1 to 3 years^9^.

In the second scenario, when a causal genetic variant has not been found in the proband, a repeated cardiac evaluation with ECG and echocardiography is recommended every 3–5 years before 10 years of age, every 1–2 years between 10 and 20 years of age and every 2–5 years thereafter in first degree relatives^9^.

The age at which genetic studies should be performed remains controversial. The American Society of Human Genetics (ASHG) published a statement on genetic testing in children and adolescents in 2015 that encouraged parents to defer predictive or pre-dispositional testing for adult-onset conditions such as HCM until at least adolescence, unless there is a specific clinical intervention during childhood^11^. It was argued that due to the complexity of genetic information, this should be offered when the patient can really understand it. Likewise, the European Society of Genetics recommends delaying the time of genetic studies in children as the genetic diagnosis can add anxiety, and promotes overprotection^12^. On the other hand, some studies have observed that mutation carriers of HCM causing mutations do not have a worse health-related quality of life or more anxiety as compared to a representative sample of children from the general population^13^. Despite these recommendations it is not uncommon that predictive genetic testing is offered to minors and even the ESC HCM guidelines suggest that it could be considered in children over 10 years-old. The arguments of those who favor early genetic testing is that it allows orientation of children sport activities and removes uncertainty to parents. Although there is no current consensus among cardiologists about performing genetic testing in asymptomatic children without signs of HCM, the child’s well-being and interests should always be taken into consideration.

Advanced echocardiographic characterization with tissue Doppler and strain imaging is useful in mutation carriers without an overt HCM phenotype. Tissue Doppler imaging (TDI) techniques have shown decreased systolic and diastolic velocities independently of left ventricular hypertrophy^14^ and could be used to plan a closer follow-up in those patients with lower TDI velocities. Likewise, strain analysis has shown a delay in untwist and unstrain rates in mutation carriers without HCM phenotype^15^. Regarding cardiac MRI, it can be useful in genotype-positive subjects with borderline ventricular thicknesses by echocardiographic analysis or when acoustic window is suboptimal. In early stages of disease, it may show structural abnormalities such as crypts, papillary muscle anomalies or septo-apical bundles, as well as focal LGE^16^.

## General lifestyle considerations for patients with hypertrophic cardiomyopathy

Most HCM patients are able to carry out normal activities without restriction, but during follow-up visits the cardiologist or general practitioner should spend some time explaining certain general indications that need to be taken into account in daily life.

### Sport

One of the patients’ main concerns is exercise and sport. Patients with HCM should generally avoid competitive sports and focus on recreational activities. Current algorithms to predict the risk of SCD in to HCM are to be applied in the general population^17^, but there is no evidence that they can be used in athletes. Thus, both the ESC and the AHA restrict intense competitive sports in HCM patients^1,10^. On the other hand, asymptomatic HCM patients with mild LV hypertrophy below 20 mm, no ventricular arrhythmia at exercise treadmill test or ECG monitoring and no family history of SCD may participate in competitive low-intensity sports such as golf or bowling^17^.

**Table 1:** Other lifestyle considerations for patients with hypertrophic cardiomyopathy. Reproduced and adapted from 2014 HCM ESC guidelines (Elliott et al. Eur Heart J. 2014;35 (39):2733–2779).^1^

Topic	General guidance
Diet, alcohol and weight	Patients should be encouraged to maintain a healthy body mass index Large meals can precipitate chest pain, particularly in patients with LVOTO. Smaller, more frequent meals may be helpful Avoid dehydration and excess alcohol, particularly in patients with LVOTO Constipation is a frequent side-effect of verapamil/disopyramide and should be managed with diet and if necessary aperients
Smoking	There are no data that show an interaction between tobacco smoking and HCM, but patients should be provided with general advice on the health risks associated with smoking and, when available, information on smoking cessation
Sexual activity	Patients should be given the opportunity to discuss their concerns about sexual activity. Anxiety and depression following a diagnosis are frequent and some patients may express guilt or fear about their genetic diagnosis and the risk of transmission to offspring Patients should be counselled on the potential effect of their medication on sexual performance In general, patients should avoid phosphodiesterase type 5 (PDE5) inhibitors, particularly when they have LVOTO
Medication	Patients should be provided with information about their medication, including potential side-effects and interactions with prescribed medications, over-the-counter remedies and other complementary therapies Where possible, peripheral vasodilators should be avoided in patients, particularly when they have LVOTO
Vaccination	In the absence of contraindications, symptomatic patients should be advised to have yearly influenza vaccination
Driving	Most patients should be eligible for an ordinary driving licence and can continue driving unless they experience distracting or disabling symptoms Advice on driving licences for heavy goods or passenger-carrying vehicles should be in line with local legislation For further advice on driving with ICD see European Heart Rhythm Association guidelines and local rules.
Holidays and travel insurance	
Education/schooling	Teachers and other carers should be provided with advice and written information relevant to the care of children with HCM In the absence of symptoms and risk factors, children should be allowed to perform low to moderate level aerobic physical activity, in accordance with advice from their cardiologist. Provision should be made for children with learning difficulties and other special needs

The recently published RESET-HCM trial observed that HCM patients with moderate-intensity training presented a statistically significant increase in exercise capacity at 16 weeks measured with peak oxygen consumption compared with usual activity^18^. Hence, it seems reasonable to recommend low to moderate intensity exercise in HCM patients in order to maintain a healthy lifestyle, but dissuade them from practicing high intensity sports until more data is available in this matter. Regarding definite mutation carriers with no HCM phenotype, the 2015 AHA/ACC eligibility and disqualification recommendations for athletes state that participation in competitive athletics is reasonable in the absence of family history of SCD related to HCM^17^. As for the ESC HCM guidelines, sports activities are allowed taking into account the underlying mutation and the type of sport activity^1^.

### Occupation and life insurance

The vast majority of patients with HCM will be able to continue with their job^1^. Some exceptions are military services, pilots, law enforcement or firefighting. For instance, in the case of the US military, HCM is considered disqualifying. If newly developed once enlisted, each case is independently assessed^19^. Each country has its own legislation regarding this issue and it is important to discuss the potential professional implications during genetic counselling in HCM relatives. Life insurance depends again on the rules that apply in different countries. In any case, HCM patients might find mores difficulties trying to find life insurances or mortgages.

### Prevention of infective endocarditis

Current European and American guidelines recommend good oral hygiene in HCM patients but not antibiotic prophylaxis in HCM patients undergoing dental procedures^20,21^.

In the past, infective endocarditis (IE) antibiotic prophylaxis was recommended for all HCM patients before invasive procedures^22^. However, in 2007, the AHA revised the recommendations and retired antibiotic prophylaxis for HCM patients due to an apparently significant morbidity associated with IE antibiotic prophylaxis therapy, and a lack of evidence supporting efficacy of antibiotic prophylaxis in IE prevention^21^. A recent study has observed that previous dental procedures and streptococcal infections are higher in IE in HCM patients compared to those with antibiotic prophylaxis indication, suggesting that these patients could benefit from prophylaxis^23^. Other issues regarding lifestyle are described in Table 1, based on the ESC guidelines^1^.

## Reproduction and contraception

### Reproduction

In most cases women with HCM tolerate pregnancy fairly well^1^. However, a recent study with data from the ESC initiated Registry of Pregnancy and Cardiac disease (ROPAC) has observed that pregnancy may not be as benign as previously believed, as major cardiovascular events were present in 23% of cases. There was no maternal mortality, but 15% presented HF and 12% arrhythmic events^24^. A pooled analysis with 408 cases has observed that HCM mortality during pregnancy is 0.5%, and the 2 reported deaths corresponded to high risk patients: one very symptomatic with LVOTO and a 30 mm interventricular septum and the other with a strong family history of SCD and evidence of ventricular tachycardia (VT) before death^25^. Thus, risk assessment before pregnancy should include a detailed medical history to confirm the patients’ functional status and a thorough physical examination to detect signs of HF, which are important risk factors for further complications in pregnant women with HCM.

Follow-up of pregnant HCM women should include an echocardiogram each trimester or earlier if new symptoms occur. As previously stated, symptomatic patients before pregnancy present a higher risk of complications and should be assessed regularly.

Beta blockers should be continued if they were used before pregnancy and have a solid clinical indication^1^, and can be initiated during pregnancy if required. In any case, monitoring of fetal growth is advised when using these drugs. Acebutolol, pindolol and sotalol present a FDA pregnancy category B and frequently used drugs such as carvedilol, metoprolol or bisoprolol a pregnancy category C, as there are no well-controlled studies in humans but potential benefits may warrant its use despite potential risks. Only atenolol is categorized as D due to an apparently increased risk of intrauterine growth retardation^26^.

Calcium channel blockers such as verapamil can also be used during pregnancy with an FDA Class C recommendation. Amiodarone, on the other hand, increases the risk of fetal thyroid toxicity, neurological adverse effects and growth retardation^27^. Thus, it should be avoided and only used if strictly necessary.

Planned vaginal delivery is the preferred choice in most of the cases. However, caesarian section should be considered in patients with severe LVOTO or severely symptomatic HF^1^. Epidural and spinal anaesthesia must be administered with caution in women with severe LVOTO to avoid vasodilatation and hypotension, thus single-shot spinal anaesthesia should be avoided. Oxytocin should also be given cautiously avoiding hypotension, tachycardia and arrhythmia. Attention should be paid to volemia, avoiding not only blood loss and preload decreases but also fluid overload after delivery with high risk of pulmonary oedema^28^.

### Contraception

Oral contraceptives are preferred to barrier methods in order to prevent unintended pregnancies, but estrogen-based pills should be avoided in patients with an increased thrombotic risk, such as women older than 35 years of age, smokers, previous history of atrial fibrillation, venous thromboembolism or HF. On the other hand, progesterone-only contraceptives increase liquid retention and are worse tolerated in women with HF. A safe alternative is the levonogestrel-releasing intra uterine device (IUD)^1^. In the event of pregnancy termination, prostaglandin F should not be administered as increases pulmonary artery pressure. Prostaglandin E1 or E2 are safer alternatives^1^.

### Reproductive counselling

Most HCM cases present an autosomal dominant inheritance, so there is a 50% chance for the offspring to carry the cardiomyopathy-causing mutation. As penetrance is not usually complete, the parents-to-be need to know that their future child may inherit a predisposition for HCM, but not necessarily will develop a HCM phenotype. Furthermore, if the child develops HCM, expressivity is variable even between members of the same family, so information regarding a specific clinical phenotype or prognosis cannot be accurately provided. With this information, parents may consider a natural conception or different methods to avoid the inheritance of the disease-causing mutation. These include adoption, gamete donation (oocyte or sperm), prenatal diagnosis techniques or preimplantation genetic diagnosis (PGD) which are subject to the legislation of each country, as well as to the internal regulations of each health system.

The two routinely used techniques for prenatal diagnosis are chorionic villus sampling or amniocentesis. In both cases, if the foetus is affected the parents have the option to terminate pregnancy if permitted in their residing country. PGD combines in vitro fertilization (IVF) and genetic analysis to test an embryo for the specific familial mutation before implantation. Unaffected embryos are selected and transferred to the mother’s uterus. During reproductive counselling, parents should be advised that there are approximately 0.5% of false negative results^29^, which implies that the disease-causing mutation will be transmitted to the offspring.

Furthermore, each country is subject to specific laws on assisted reproduction that the doctor must know to offer reproductive advice to patients.

## Prognosis

### General prognosis of HCM patients

According to its relatively high prevalence (1/500 in general population, the highest for an inherited heart disease) and the low global incidence of complications, the general prognosis of a HCM patient is generally good and it is commonly regarded as a benign disease^30^. Thus, two thirds of patients with HCM present a normal life span without significant morbidity^30^. A recent series reviewed HCM mortality rates and described a 0.7%/year HCM-related mortality rate and a 1.1%/year rate for HCM-unrelated causes of death^31^. Most of the herein commented complications arise in patients at high risk of SCD or HF, many of these with severe LVOTO.

### SCD prevention in HCM patients

As previously presented in a specific chapter, SCD risk stratification in HCM patients is evolving and remains challenging. The HCM-risk score adopted by ESC HCM Guidelines in 2014^1^ may still be improved in the future to maximize its accuracy. Some interesting points in this field are commented on hereafter.

### Prognostic factors in HCM

#### Age

Age has historically been warranted as a modulator of the outcomes in HCM patients. Notably, clinically stable patients who achieve the age of ≥60 years experience a subsequent clinical course with a particularly low SCD event rate (0.2%/year)^32^. A younger age at diagnosis has been thought to portend a reduced life expectancy and limited effective treatment options in the literature. Two contemporary papers have shed some light in the current scenario with updated management options. In both, idiopathic HCM in children exhibited a good 5-year survival of 94–97% for those diagnosed after 1 year of age and similar to middle-aged adult HCM patients^33,34^ which is reduced down to 82% for those diagnosed before 1 year of age^33^.

Considering HCM phenocopies in children and adolescents <18 years, similar outcomes were pointed out for those with neuromuscular disorders (5-year survival of 98%). On the other hand, children with HCM associated with an error of metabolism or malformation syndrome manifest the disease at a younger age, and had low 5-year survival rates (42% and 74%, respectively)^33^.

Actually, among HCM patients from 7 to 30 years old, the HCM-related mortality was as low as 0.54%/year, similar to that found in patients aged 30-59 and ≥60. These findings were explained by high rate of nonfatal HCM events aborted with contemporary treatment interventions in the pediatric population (>2-fold than that of older patients), which finally yielded a low incidence of SCD and HF HCM deaths of 0.39%/year and 0.17%/year, respectively^34^.

Finally, it has been repeatedly stressed that the ESC risk score model proved highly ineffective for identifying patients at the greatest SCD risk^34^. Endorsed by a current meta-analyses, among pediatric HCM population only 4 ‘major’ risk factors that have been shown to be statistically associated with increased risk of death in at least 2 studies (previous adverse cardiac event, non-sustained ventricular tachycardia (NSVT), syncope and LVH)^35^.

#### Apical aneurysms

Apical aneurysms are found in 5–8% of HCM patients, who exhibit a poorer prognosis^36,37^. Cardiac MRI emerges as the most useful technique for its diagnosis (40% can be missed with other imaging methods) and ventricular arrhythmia substrate analyses (LGE in the aneurysm rim)^36^. It has been suggested that apical aneurysms should be considered a new risk factor for SCD on the basis of a high rate of HCM-related deaths combined with life-saving aborted disease-related events (6.4–10.1%/year, more than 3-fold greater than the 2.0%/year event rate in 1,847 HCM patients without aneurysms, *p* < 0.001)^36,37^. Moreover, apical aneurysms also increase morbidity and could prompt physicians to initiate anticoagulation therapy due to an incidence of stroke of 1.1%–4.0%/year^36,37^. Anticoagulation due to clot identification in 15% of these patients prevented embolic complications in 4 years of follow-up and radiofrequency ablation of monomorphic ventricular tachycardia yielded a high success rate^36^.

#### Non-sustained ventricular tachycardia (NSVT)

NSVT are registered during holter monitoring in 20-30% of HCM patients^1^ and were included in the current ESC proposal of HCM risk stratification as ≥3 consecutive ventricular beats at ≥120 BPM lasting <30 seconds. Although it was acknowledged that no evidence supported a particular frequency, duration or rate in risk modulation, more recent publications on extended monitoring, confirmed that NSVT was independently associated with ICD-treated ventricular arrhythmias, but only when their rate was >200 beats per minute, when they lasted >7 beats, and when repetitive (adjusted hazard ratios 6–15, *p* < 0.05)^38^. Apart from resting NSVT, ventricular arrhythmias may also be exercise-induced. In this case, they appear to be closely related to myocardial fibrosis^39^. The promising results from the London cohort linking exercise induced-arrhythmias and a poor prognosis^40^ have not yet inspired more papers either to confirm or to argue their conclusions. In that series 27/1380 patients exhibited NSVT or ventricular fibrillation (VF) during exercise and those arrhythmias were associated to more severe hypertrophy, larger left atria, male sex and death or cardiac event (3.73-fold increase in risk of SD/ICD discharge), the latter also in the multivariate analysis (hazard ratio 3.14, 95% CI [1.29–7.61])^40^.

#### Myocardial fibrosis

With respect to prognosis in any cardiomyopathy the presence and magnitude of myocardial fibrosis (or LGE in cardiac MRI) has been a trending topic in the past 10 years. Particularly in HCM patients, it appears clear that the percentage of patients with LGE and its quantity (grams and percentage of LV myocardial mass with LGE) correlates with systolic impairment and HF (end-stage HCM)^41^. Regarding the arrhythmic risk, in a recent metaanalysis with pooled data the amount of LGE remained independently associated with SCD risk (adjusted hazard ratio 1.4 for every 10% increase in LGE of LV mass, and adjusted hazard ratio of 1.6 for 15% LGE)^42^. Based on these data, it may be reasonable to consider that patients with HCM and extensive LGE (≥15–20% left ventricular myocardium) present an increased risk, regardless of other high-risk features, with implications on management strategies including ICD implantation. Extensive data can be found in the chapter regarding cardiac imaging.

The blunted perfusion reserve of HCM patients (related to microvascular dysfunction and small vessel disease) precedes the development of myocardial fibrosis and systolic dysfunction by years^43^, so it could be an early marker of poor prognosis. Thus, early identification of microvascular flow abnormalities with PET studies represents an opportunity for pharmacological prevention of disease progression^44^ since once fibrosis has been established in HCM patients, little can be done from a curative therapeutic approach.

#### Genotype

Genetics may also have an influence in the general HCM prognosis. Patients with sarcomeric protein variants (nearly 44% of HCM patients) are characterized by younger age and higher prevalence of family history of HCM, family history of SCD, asymmetric septal hypertrophy, greater maximum LV wall thickness and an increased incidence of cardiovascular death (all *p* values<0.02)^45^. Besides the mutated gene, the literature underlines that also the number of genetic hits may be relevant. Thus being a triple mutation carrier, although rare (0.8%), conferred a remarkably increased risk of end-stage progression (14-fold increase) and ventricular arrhythmias (100% were deemed at high risk of SCD and were ICD carriers)^46^. Although the presence of mutations have been associated with worse prognosis that the absence of a positive genetic test, recent data have demonstrated that individual mutations currently do not allow to establish prognosis in the majority of patients^47^.

#### Diastolic dysfuntion

Diastolic dysfunction promotes adverse remodeling over time in HCM patients. It has often (but not always) been related to HF development. This observation is based on several studies reporting a 100% of impaired LV filling pattern in non-obstructive HCM patients with HF (43–50% with additional left ventricular ejection fraction (LVEF) <50% and 40-100% with restrictive pattern)^48,49^. Strikingly, the most adverse LV filling pattern alteration, a restrictive pattern, was depicted in 5.9% of HCM patients at initial evaluation and in 9.2% during follow-up^50^. Among these patients HCM behaved aggressively with a 6-fold increase in risk of developing end-stage HCM, a 0.95%/year of SCD or appropriate ICD interventions, and a 3.2%/year of HCM-related death or heart transplantation^50^. Thus, the restrictive filling pattern was a strong and independent marker of increased risk compared to patients without restrictive filling (hazard ratio for SCD 3.5, and for a wider endpoint also including heart transplantation, resuscitated cardiac arrest, and appropriate ICD intervention 5.1, *p* < 0.05 both)^50,51^.

Finally, not only the LV restrictive filling pattern but also the RV restrictive physiology appears to have significant predictive value in HCM, regardless of the presence of other detrimental risk factors^52^.

#### Left ventricular ejection fraction

Systolic dysfunction is regarded as a clear marker of adverse prognosis in HCM patients (see *Heart failure* in the complications section below).

#### Autonomic dysfunction

Autonomic dysfunction in HCM patients precedes systolic dysfunction and so may play a role in its genesis, can be depicted by nuclear medicine studies assessing the pre-synaptic level and could represent a future therapeutic target to improve outcomes^44^.

#### Left ventricular outflow tract obstruction

A number of studies have proved a significant association of the LVOT maximal gradient with an increased risk of SCD, as cited in the current guidelines^30^. However, less evidence supports the role of a provoked LVOT gradient and it appears that, in keeping with the same rationale, provoked LVOT gradients <30 mmHg, 30–90 mmHg and >90 mmHg could identify HCM patients of low-, intermediate- and high-SCD risk^53^.

#### Pulmonary hypertension

Pulmonary artery systolic pressure (PASP) over 36 mmHg is one of the features of end-stage HCM^40,54^. It is present in nearly 40% of HCM patients being its predictor factors older age, female sex, AF and class II-IV NYHA class^54^. More interestingly, it was identified as the only independent predictor of all-cause mortality except in patients with obstructive HCM who underwent septal reduction therapy (in non-obstructive HCM hazard ratio 1.59 per 10 mmHg PASP increase; in obstructive HCM without septal reduction therapy hazard ratio 1.15 per 10 mmHg PASP, both *p* < 0.05)^54^.

#### ECG repolarization

Inconstantly, exercise-induced microvolt T-wave alternans has been associated to ventricular arrhythmia susceptibility (as a subrogate marker of an increased risk in HCM patients)^55,56^.

#### Biomarkers

Several plasmatic biomarkers have been analyzed aiming to predict prognosis in HCM patients. A high hsCRP concentration associated to more adverse events (>3.0 mg/L versus <1.0 mg/L: adjusted hazard ratios for individual adverse events 4–11-fold, *p* < 0.05) and it was replicated when considered as a continuous variable (adjusted hazard ratios for individual adverse events 1.06–1,20, *p* < 0.05)^57^. NT-proBNP concentration independently predicted all-cause mortality or cardiac transplantation being a serum concentration of ≥135 pmol/L associated with an annual event rate of 6.1%^58,59^. Although NT-proBNP has repeatedly been identified as a significant independent predictor of HF and transplant-related deaths there is not total agreement in the literature concerning its accuracy to predict SCD or appropriate ICD shock^51,58,59^.

Several metalloproteinases (MMPs) are increased in the serum of HCM patients, such as MMP-9 and MMP-3, respectively associated with LGE and increased arrhythmic risk^60^. Since aldosterone is known to promote MMP expression and is elevated in HCM patients it has been repeatedly identified as a potential therapeutic target^44^. Also Copeptin (the stable C-terminal part of pro-arginine vasopressin, also termed antidiuretic hormone) remained as an independent predictor of HF and adverse cardiac events in multivariable logistic regression analysis on a small HCM cohort followed during 24 months^58^.

Scarce yet interesting evidence supports the role of the soluble suppression of tumourigenicity (sST2) in HCM risk stratification due to the presence of higher levels in HCM patients with NSVT^61^. Increased transforming growth factor-beta levels, however, identified patients with higher risk of developing postoperative AF after myectomy but not major adverse cardiac events^62^. Interleukin-6, tumour necrosis-alpha and soluble Fas ligand have been associated to fibrosis in HCM patients, and so indirectly linked to HCM prognosis^44^.

Finally, regarding the attractive role of microRNAs as key modulators of gene expression, the circulating levels of miR-29a were found to be up-regulated in HCM patients, correlating with both myocardial hypertrophy and fibrosis, and indirectly suggesting a potential prognosis value for this microRNA^63^.

**Table 2:** Clinical characteristics of different HCM phenotypes. Reproduced and adapted from Olivotto I, et al. Circ Heart Fail. 2012;5(4):535–46.^41^

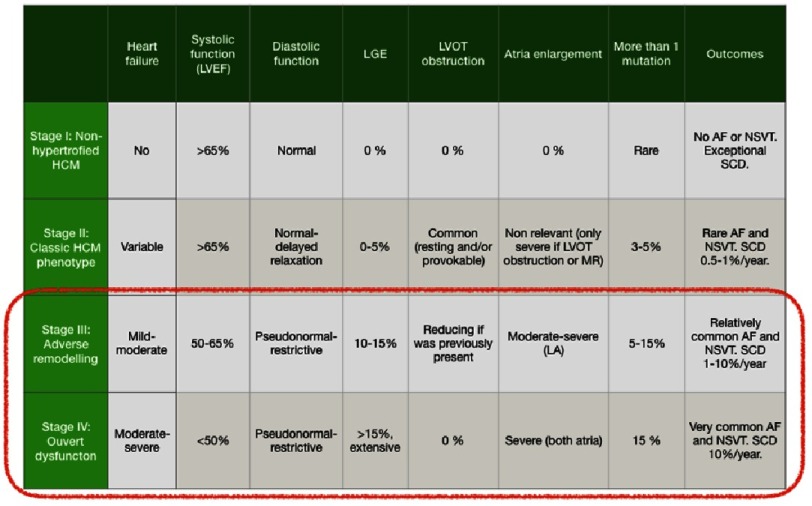

### Clinical complications of HCM

#### Heart failure

A life-long remodelling process takes place within the myocardium of HCM patients which leads to clinical progression and may culminate in the so-called end-stage or burnt-out phase in 5–7% of patients, at any age, and with a 10-fold increase in SCD estimates (Table 2)^41^. Four stages of disease have been proposed to define the natural history of the disease (i) ‘Non-hypertrophic HCM’, characterized by the absence of LVH in mutation carriers (the occurrence of live-threating ventricular arrhythmias is exceptional); (ii) ‘Classic HCM phenotype’, defined as fully developed hypertrophy with hyperdynamic left ventricle, often with LVOTO but without significant fibrosis; (iii) ‘Adverse remodelling’ (15% of HCM patients), where already unfavourable structural modifications have occurred (extensive LV fibrosis and LVEF in the low normal range of 50–65%), with preserved clinical and hemodynamic balance; (iv) ‘Overt dysfunction’ (5–7% of HCM patients), characterized by severe functional deterioration of the LV (defined as overt systolic dysfunction and/or restrictive pathophysiology), extreme LV fibrosis and atrial dilatation, haemodynamic decompensation and adverse outcome^41,49^. Treatment with HF drugs, aggressive management of AF and prevention of SCD in stages III and IV should be taken into account and the promising role of ranolazine could also open new doors to improve outcomes at this point^41^.

#### Atrial fibrillation

An important proportion of HCM patients develop any kind of new onset atrial fibrillation (AF), namely 17–22% of patients during 9–22 year follow-up period, 2%/year^65,65^. The timing and frequency of paroxysmal AF episodes are unpredictable in HCM patients, with an average 2-year interval between the first and second symptomatic episode, but progressing to permanent AF uncommonly (26%)^31^. AF has been generally associated with increased morbidity and mortality in HCM patients. However, two recent studies showed opposite conclusions at this point: one yielded a 2-fold increased risk of cardiovascular death at 10 years in HCM patients with AF^64^ whereas the other pointed out that with the current strategies AF is not a major contributor to HF or a cause of arrhythmic sudden death, assuming that AF usually appears to represent a secondary event, a disease marker, or largely an innocent bystander to clinical events, rather than a primary cause^31^. Notably, the recruitment and follow-up periods were significantly different in both studies, from 1986–2008 the former^64^ and 2004-2014 the latter^31^ which may have influenced the treatment strategies employed. In the most contemporary series, no differences regarding AF development were found in all cause or HCM-related mortality (0.7%/year), and progression of HF symptoms from class I/II to III/IV at 5 years (approximately 5%, and without differences when considering paroxysmal or permanent AF)^31^.

The occurrence of new onset AF and the presence of permanent versus paroxysmal AF was associated with female sex, age, left atrial diameter, and NYHA class, whereas the new onset was additionally associated to hypertension and vascular disease and the progression to permanent AF to LVEF, larger LV end-diastolic cavity size and less common resting LVOTO or myectomy^31,64^. On multivariable analysis, the only independent predictors for development of AF were younger age at HCM diagnosis, larger LA, and lower LVEF^31^.

Regarding the most frequently used treatments, oral drugs were unsuccessful to maintain^64^ sinus rhythm and to restore it, same as Maze procedures^31^. Regarding catheter ablation for AF treatment, long-term outcomes was worse in patients with apical HCM, as compared to controls, but was similar to patients with septal HCM (50% free from AF/atrial tachycardia with a 44.7 ±30.8 months) and LA diameter was an independent predictor for recurrence^66^.

##### Stroke.

Stroke is considered the most disabling consequence of AF in HCM patients, and under the currently available management it presents with a wide range in 6–28% of these patients being 11% of them fatal strokes^31,64,67^. Given that HCM patients with AF usually would not fulfill anticoagulation criteria by CHADs scoring, anticoagulation is always recommended, irrespective to CHADs score system^1,31,68^. Although little evidence supports the use of direct oral anticoagulants, initial evidence supports that at least equals anti-vitamin K management with a higher treatment satisfaction among patients^69^.

##### Infective endocarditis.

The incidence of IE among HCM patients has been described to be 18 to 28 times higher than in the general population being the LVOTO and enlarged LA its reported risk factors^70^. The two largest retrospective cohorts (*N* = 30 and *N* = 34) pointed out the non-determinant presence of LVOTO and similar rates for surgical intervention (37–42%) but differed in the 1-year mortality (7% versus 42%), aortic and mitral valve involvement (similar versus predominantly mitral), and embolic complications (33% versus 18%).^23,71^ Although since 2009 antibiotic prophylaxis is no longer recommended by the ESC in HCM patients^20^, the very low incidence of prophylactic complication and the high risk of complications in an HCM patient with IE still generate debate and warrants reconsideration of the balance of risks/benefits in this scenario. Thus, some dedicated clinics currently recommend IE antibiotic prophylaxis.
